# Episodic Binding and Retrieval in Sequences of Discrete Movements – Evidence from Grasping Actions

**DOI:** 10.5334/joc.234

**Published:** 2022-07-14

**Authors:** Marie C. Beyvers, Iring Koch, Katja Fiehler

**Affiliations:** 1 Department of Experimental Psychology, Justus Liebig University Giessen, Germany; 2 Department of Cognitive and Experimental Psychology, University of Aachen, Germany; 3 Center for Mind, Brain and Behavior (CMMB), University of Marburg and Justus Liebig University Giessen, Germany

**Keywords:** Action, Action and perception, Cognitive Control

## Abstract

In everyday life humans are confronted with changing environmental demands. In order to act successfully and achieve intended goals, action control is required. A recent approach, the Binding and Retrieval in Action Control (BRAC) framework, attempts to provide an overarching perspective on action control. Based on basic principles such as binding and retrieval, findings from several experimental paradigms could be integrated. However, the focus so far has been on rather artificial paradigms involving very simple motor response requirements, like finger lifting or button presses. We aimed to extend the BRAC framework to more complex movements consisting of a sequence of several discrete actions. Participants were asked to grasp and lift an object with an uneven mass distribution. Object features, like mass distribution and position, were either kept constant on a global level or varied in a pseudorandomized manner. When both object features were kept constant, participants were able to adjust their grasp so that it resulted in a more stable lift and less object roll. Further, with randomly mixed object features, we found best task performance when both object features were completely repeated from one trial to the other. These results suggest that tasks with more complex movements are capable of reflecting principles of action control as defined by the BRAC framework. This offers the possibility to test these principles in even more complex and ecologically relevant paradigms to improve our understanding of everyday life actions.

## Introduction

Humans continuously perform actions to interact with their environment to produce desired perceptual action effects (Dignath et al., 2014; Pfister et al., 2014). These actions move along a continuum from simple actions, such as flipping a light switch, to more complex action sequences, such as brewing our morning coffee. And even if some actions seem trivial in everyday life, the underlying processes are far more complex. For example, when reaching for our cup of coffee in the morning, many factors must be considered for this action to be successful: the location of the cup, its weight, as well as the nature of the handle and its surface structure. Prior experience or visual cues can help to identify these factors; however, they are always associated with some degree of uncertainty (Faisal et al., 2008; Schultheis & Rothkopf, 2021). Especially when actions become more complex and occur in situations involving change or uncertainty, they must be adaptive and flexible to respond to these changing environmental demands (Frings et al., 2020). This poses increased demands on processes of action planning and control, which are required to perform such complex movements in order to achieve the intended goals.

Action control is driven by different aspects of cognition, like perception, attention, memory, and motor planning (Frings et al., 2020). To date, a wide variety of theories of action control, such as S-R Binding Approaches to Memory (Henson et al., 2014) or Theory of Event Coding (Hommel et al., 2001), have been developed to explain the results of specific paradigms. Some of the most commonly used paradigms are task switching (Koch et al., 2018), negative priming (Frings et al., 2015; Mayr & Buchner, 2006), or stimulus-response binding (Frings et al., 2007; Hommel, 1998). Theories of action control have several commonalities (Frings et al., 2020). They usually rely on tasks using a sequential trial methodology – the processing of information at one (prime) trial influences the processing and reaction at a subsequent (probe) trial. Binding approaches propose that in the process of motor planning, cognitive mechanisms are often influenced by specific aspects of previous actions. These situational action aspects are coded as elementary features and are thought to be integrated into a common episode or “file” (e.g. Hommel, 1998, 2004; Treisman, 1996). Such an episode is stored in memory and can later be reactivated (i.e., retrieved) through feature repetition. Although there are many commonalities among the different theoretical approaches, paradigm-specific theories have been repeatedly put forward instead of a unified theory.

A recent approach to action control, the Binding and Retrieval in Action Control (BRAC) framework, provides a general and comprehensive theoretical framework for various experimental outcomes from different paradigms (Frings et al., 2020). BRAC focusses on two core processes of action control: feature binding and feature-based event-file retrieval. Following the sequential logic of many experimental paradigms, features of the stimulus environment, a response in that environment, and its subsequent effects are thought to be bound together in an event-file. These event-files can be multisensory, for example, when visual, tactile, and auditory features are combined. If any feature is repeated in a subsequent trial, this will trigger automatic retrieval of the previous event-file from memory. Depending on the requirements of the respective trial the retrieval may result in performance benefits or costs. For example, in task switching paradigms (e.g. Koch et al., 2018), two tasks are performed alternately (e.g. odd/even judgement of numbers vs. vocal/consonant judgement of letters) by using the same keys to make the different judgments. If the task changes, a deterioration in the performance, usually reflected in longer reaction times, can be observed in comparison to a repetition of the task. The hitherto established explanation for this switch cost embraces the interference of the new task with the persistent inhibition of the competing task (see Koch et al., 2010, for a review) or the persistent activation of the previous task, impairing performance (see Koch & Kiesel, 2022, for a comprehensive review of theoretical accounts). Importantly, task switch costs could also be explained by the integration of both the stimulus and task in the prime trial (Frings et al., 2020; Koch et al., 2018). Upon stimulus repetition in the probe trial, the previous task is automatically retrieved, leading to interference in case of a required task switch.

Using the principles described in the BRAC framework, several other effects commonly encountered in the study of action control can be addressed as well. One aspect is the global expectation of consistency of a task, also called *mixing costs*. That is, if a task is performed several times in consecutive order, performance is often better than if the task or features of the action change from one occasion to the other (Los, 1999; Philipp et al., 2008; Rubin & Meiran, 2005; Steinhauser & Hübner, 2005). When switching between different tasks is required or a task involves mixed features, additional processing is usually needed to switch between the required actions relative to single-task conditions, leading to mixing costs (for reviews see Koch & Kiesel, 2022; Kiesel et al., 2010).

The BRAC framework can also account for results of other paradigms for the study of action control (for an overview see Frings et al., 2020). For instance, it could account for *partial repetition costs* (Hommel, 1998). That is, when confronted with sequences of mixed stimulus-response combinations, participants generally perform better when the current stimulus and response features are either completely repeated or when no feature is identical (Hommel, 1998, 2004). Thus, performance is most likely to be impaired when one feature repeats while another changes. This leads to partial repetition costs, suggesting that previously linked feature bindings are retrieved, which in case of partial repetitions create a conflict between the retrieved action and the action that is actually required (Frings et al., 2020). The retrieved action must be suppressed and therefore results in a relative impairment compared to cases in which all features or no feature are repeated. Overall, the BRAC framework is intended to integrate action-related phenomena from different research areas. This framework should allow for a broader application even beyond the scope of typical cognitive studies on action control.

As BRAC is aimed at providing an overarching perspective on action control, the described core principles of binding and retrieval should be transferable to different experimental paradigms. This transfer has been done to some extent by focusing on rather artificial paradigms using very simple motor response requirements. In these paradigms, stimuli are usually presented on a computer screen and prompt for a button press as the required action. Furthermore, features in these simple paradigms are often clearly perceivable at the beginning of each trial. If the principles postulated in the BRAC framework are universal (as claimed), they should also be applicable to actions involving movements of higher complexity (i.e., more degrees of freedom). Many of our everyday-like actions are characterized by sequences of several discrete movements, e.g. when grasping a cup to drink or preparing a sandwich. Additionally, in such action sequences, not all object features are always continuously perceivable but can sometimes only be experienced through interaction with the object, e.g. when lifting an opaque container filled with liquid. The goal of this study was to test whether the BRAC framework can be generalized to more complex types of actions.

In this study, we examined a situation that required a grasp-to-lift movement, which can be characterized as a sequence of discrete, goal-directed movements, comprising transport, grip, lift, and place phases. In particular, in each experimental trial, participants had to grasp an object with an uneven mass distribution and lift it as straight as possible. Previous studies showed that in such a task participants were more likely to lift the object straight up when the object’s mass distribution did not change compared to a situation when it changed in a random manner (Broda et al., 2020; Voudouris et al., 2019). This could be related to the fact that participants tended to grasp the object in the same way as in the previous trial, even though different grasp configurations were possible (Dixon et al., 2012). Moreover, when grasping to lift an object with an unknown mass distribution, participants tended to use a grip that would stabilize the object during lift, expecting it to have the same mass distribution as experienced in the previous trial (Lukos et al., 2013). This previous trial effect even occurred when participants were aware that the mass distribution of the object would randomly change from trial to trial and thus seems to be rather automatic. Action planning in the context of object interaction generally relies on dynamic object representations acquired through previous object interactions (Schneider et al., 2019). In this regard, participants prepare for an action by retrieving features of the action from memory and the object to be grasped represents a decisive cue for this retrieval from memory (Dixon et al., 2012). Overall, there is some evidence that cognitive aspects of action control, like binding and retrieval, also have a substantial influence in more complex actions, but it remains unclear to what extent the BRAC framework can be applied to sequences of discrete actions and what are the similarities and differences compared to effects in single discrete actions (e.g., a single button press).

The aim of this study was to test the principles of the BRAC framework for a sequence of discrete, goal-directed movements which were characterized by a recognizable start and end point (cf., Fitts & Posner, 1967). Participants performed an unimanual object interaction task. They grasped and lifted an object with an uneven mass distribution from two different positions in a sequential order. To examine both mixing costs and partial repetition costs, the trial sequence contained blocks in which object features were either kept constant or changed in a pseudorandomized manner. As participants always viewed the same objects and accomplished the same task, we expect a memory retrieval of all features associated with the object from a previous event file, even if not all object features (mass distribution) are visible at the beginning of each trial. This is comparable to a simple button press experiment where, for example, a circle is displayed at one of two positions on a screen. In this example, the first step would be to respond to the position of the circle by pressing a button. After that, the circle would turn red or blue, which would require another button press response. If the circle is displayed on the right side and then turns blue, it would be assumed that these two features are bound to a common episode. If the circle is presented again on the right side in the next trial, according to the BRAC framework one would assume a bias to respond to the blue color, even if it is not yet visible. If the circle then appears red, a drop in performance would be expected (partial repetition costs). Here, we applied the same experimental logic to more complex movements where features are experienced through object interaction.

Since action sequences involve a longer and more complex temporal structure than single, discrete actions such as button-presses, reaction times might be less sensitive to capture binding and retrieval processes. For this reason, we focused on the kinematic aspects of motion, like the placement of the fingers when grasping an object (digits’ separation) and the tilting of this object (object roll), which have already been shown to be strongly influenced by the general movement context and the feature configuration of the previous trial (Broda et al., 2020; Lukos et al., 2013; Voudouris et al., 2019). By examining digits’ separation and object roll we were able to test the BRAC framework for motor planning and motor control processes, respectively. Recent studies also investigated response features other than reaction time in the context of binding and retrieval, e.g., percentage of errors or applied force (Pfister et al., 2022; Varga et al., 2022). If there are comparable effects for sequences of discrete actions as for single discrete actions, we expect better performance if (a) object features are constant on a global level, (b) object features are completely repeated within a situation of randomly mixed object features, and (c) no feature at all is repeated within a situation of randomly mixed object features. These results would underline that binding and retrieval processes also play an important role in more complex action sequences and that the BRAC framework can be applied in this context as well. This would indicate that processes of action planning and action control operate on a common, overarching basis, which in turn allows results from different branches of action research to be compared and merged to provide a better understanding of everyday life actions.

## Methods

### Participants

In total, 40 participants completed the experiment. Due to a technical problem during data collection, data from eight participants were excluded, resulting in a total sample of 32 participants (26 f, 6 m, *mean_age_* = 23.88 ± 4.08 years). In addition, we had to exclude one additional participant from the kinematic data analysis (digits’ separation) due to data loss. Due to the lack of a suitable independent data set, we did not perform an a-priori power analysis to calculate the required sample size. Instead, we set the sample size to at least 28 participants that would allow us to detect large effects with a power of 0.95 and effect sizes of *d_z_* > 0.8 (corresponding to 19 participants for t-tests) and *η*^2^*_p_* > 0.14 (corresponding to 28 participants for ANOVAs; see section Data Analysis). Participants were all right-handed, as assessed by the German translation of the Edinburgh Handedness Inventory (Oldfield, 1971; *mean* = 90, *range* = 60–100).

### Apparatus

**Figure 1:**
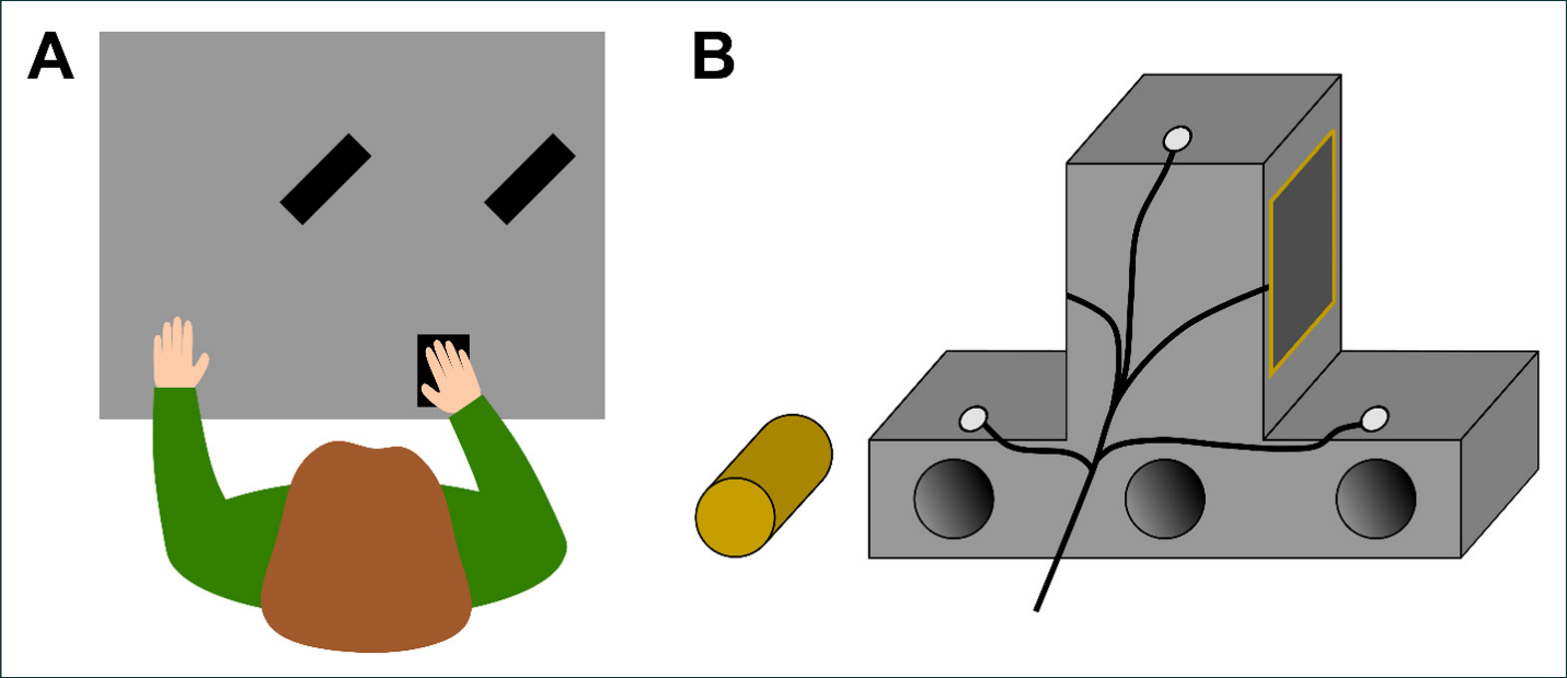
Experimental setup. **(A)** Top view of the setup with the right index finger and thumb resting on the start position, and both object positions (black rectangles). **(B)** Illustration of the backside of the inverted T-shaped object with the three infrared markers (white circles) and the touch sensors fixed to the grasping area of the object. The three tubes in the object’s base were never visible to the participant. The brass mass is depicted to the left of the object.

Participants were seated in front of an 117 × 80 cm table (Figure 1A). A small keypad (12.5 cm × 8 cm) was placed at the edge of the table approximately aligned to the right shoulder of the participant. They had to grasp and lift one of two inverted T-shape objects (Figure 1B). At the backside of the lower part of the objects, invisible to the participants, three tubes were distributed laterally. A cylindrical piece of brass (116g) was inserted in one of the tubes, creating an asymmetric object *mass distribution* (*MD*). One of the objects contained the brass mass on the left, the other one on the right side. The total mass of the objects, including the brass, was 270g each. To prevent participants from seeing in which tube the experimenter inserted the brass prior to each trial, participants wore liquid-crystal shutter glasses (PLATO, Translucent Technologies, Toronto, Canada) throughout the whole experiment. On each object, two touch sensors (4.37 × 4.37 cm; Interlink Electronics Inc., Westlake Village, CA, USA) were mounted on the grasping sides of the upper part of the object. The objects were presented at one of two *object positions* (*OP*) about 33cm from start button, which were about 27cm apart (see Figure 1A). The movement of the participant’s hand and the object were recorded with an Optotrak Certus motion tracking system (Northern Digital, Inc., Waterloo, ON, Canada) at 100 Hz. Therefore, infrared markers were attached to the fingernail of the participants’ right thumb and index finger and in a triangular arrangement on the backside of the objects. It was ensured that all markers could be correctly detected by the camera system and that cables did not restrict the participants’ movement.

### Procedure

The experiment consisted of a blocked sequence of trials differing in the arrangement of the two object features: MD and OP, each on the right or left side. Object features could either repeat (MD+, OP+) or change (MD-, OP-) from trial to trial. Four constant blocks were presented at the beginning and the end of the sequence, in which MD and OP were kept constant (MD+ & OP+, 2 × 4 × 22 trials) and thus there was always a complete repetition of both factors in each of the four constant blocks. The first of the four constant blocks was counterbalanced between participants. The order of the other three constant blocks was randomized. At the beginning and end of the sequence, the constant blocks were presented in the same order for each participant. In between these constant blocks, a longer mixed block was presented (360 trials). This mixed block was designed to prevent participants from predicting the configuration of factors of the upcoming trial (Gellermann, 1933) and to ensure that all possible combinations of consecutive MD and OP occurred equally often during the block. Thus, the mixed block contained complete, partial, and no repetition of both factors with respect to the previous trial. Overall, the sequence contained a total of 536 trials.

Each trial started with the participant pressing the start button on the keypad with their right index finger. The view of the participants was blocked by using shutter glasses. The experimenter inserted the brass cylinder into the left or the right tube of one of the objects, placed it on one of the two positions and started the trial. After the shutter glasses turned transparent, an auditory signal indicated the start of the movement. Participants were instructed to reach out and grasp the object with their right thumb and index finger (precision grip) at both sides of its upper part, where the touch sensors were attached. They should lift the object at a natural speed straight up for about 15 cm, place it back on the table and return their finger to the start position. Participants had five seconds to perform the whole movement and return to the start button. After the movement was finished, the shutter glasses turned opaque again. Before starting the experiment, participants performed nine practice trials to familiarize with the task and the object weight. For those trials, the brass cylinder was inserted into the center tube of one of the objects. During practice the object was randomly placed on one of the two positions.

### Data analysis

The main goal of the experiment was to examine the key assumptions of the BRAC framework for more complex movements consisting of a sequence of several discrete, goal-directed movements. The two considered factors (MD & OP) act together from the point where participants grasp the object. To analyze motor planning and motor control, the influence of the previous trial’s MD and OP on a) the positioning of the digits at object contact (digits’ separation) and b) the maximal object roll during lifting were examined, respectively. In order to determine the relevant kinematic measures for each trial, first the speed of the hand and of the object were calculated by numerical differentiation of the mean position of the markers on both digits and on the object, respectively. These two speed vectors were dual-pass filtered using the MATLAB filtfilt function with a 2nd order lowpass butterworth filter and a cutoff of .30. We determined the moment of object contact with the use of the touch sensors as the first timepoint when one of the digits came into contact with one of the sensors. The base value for *digits’ separation* was defined as the mean vertical distance between the two digits within the first 100ms starting from the moment of object contact, with positive values indicating that the thumb was placed higher than the index finger. The base value for *object roll* was defined as the absolute maximal tilt angle within the first 250ms after the object was lifted.

To investigate whether kinematic behavior was influenced by MD and OP experienced in the previous trial, different configurations of consecutive factor combinations were defined: MD+ involved trials with consecutive mass distributions that were identical (LL, RR), MD- involved trials with different consecutive mass distributions (RL, LR), OP+ involved trials with consecutive object positions that were identical (LL, RR), and OP- involved trials with different consecutive object positions (RL, LR). Further, the influence of the two different block types within the sequence was considered by averaging the base values of the two kinematic variables within these blocks and the different configurations. Within the constant block, only MD+ and OP+ occurred; within the mixed block, all four combinations of MD and OP were presented (MD+ & OP+, MD+ & OP-, MD- & OP+, MD- & OP-). Since there seems to be a tendency to place the digits in such a way that the MD experienced in the previous trial is compensated (Lukos et al., 2013; Voudouris et al., 2019), calculating the mean values of digits’ separation for the different configurations would negate the actual effect. Therefore, the average digits’ separation of each participant in trials with left MD were subtracted from the respective value in trials with right MD. Under the assumption that participants position their index finger higher than the thumb when they expected the weight to be on the right and lower than the thumb when they expected the weight to be on the left (Lukos et al., 2013), the resulting index value should be less than 0 when the weight position is repeated and greater than 0 when the weight position is changed. Thus, a negative index value indicates a better performance, as in this case participants positioned their fingers favorable to the distribution of the weight.

To check whether the index values for digits’ separation differed from zero, we conducted one sample t-tests against zero separately for each condition. To compare constant and mixed parts of the sequence and to examine whether the general expectation regarding the change in both features had an influence on the task, we conducted one-tailed paired samples t-tests on digits’ separation and object roll as dependent variables. We compared the constant blocks with the mixed block trials in which both MD and OP were repeated compared to the previous trial, assuming that both digits’ separation index values and object roll to be lower for the constant parts. To examine the influence of the two factors on performance in the mixed parts of the sequence, we conducted a 2 × 2 within subjects ANOVA on digits’ separation index values and object roll as dependent variables with the factors previous MD (MD+, MD-) and previous OP (OP+, OP-). In addition, we calculated the correlation between digits’ separation and object roll across all condition (Pearson’s r) to test if more efficient grasping of the object (negative digits’ separation) is indeed associated with less tilting of the object (lower object roll). All statistical analyses were carried out with JASP (Version 0.14.1). Significant interactions were inspected with post-hoc t-tests, Bonferroni-corrected for multiple comparisons (α = .008). Effect sizes are described as partial Eta squared (*η*^2^*_p_*) for ANOVAs and Cohen’s d_z_ for t-tests.

## Results

### Digits’ separation

First, we analyzed digits’ separation as a measure of motor planning processes. We found mean index values below zero for the constant condition (MD+OP+, *t*(30) = –12.97, *p* < 0.001, *d_z_* = –2.33) and the condition where both features were repeated within the mixed block (MD+OP+, *t*(30) = –2.75, *p* = 0.01, *d_z_* = –0.49). This indicates that participants positioned their fingers favorable to the distribution of the weight and thus were able to achieve better task performance. The condition where MD was repeated and OP not within the mixed block elicited a mean index value not differing from zero (MD+OP-, *t*(30) = –0.27, *p* = 0.79). Further, we found mean index values above zero for both conditions where MD was not repeated within the mixed block (MD-OP+, *t*(30) = 7.83, *p* < 0.001, *d_z_* = 1.41; MD-OP-, *t*(30) = 6.63, *p* < 0.001, *d_z_* = 1.19), indicating that participants positioned their fingers in anticipation of a repeated MD that did not occur, resulting in worse task performance.

**Figure 2:**
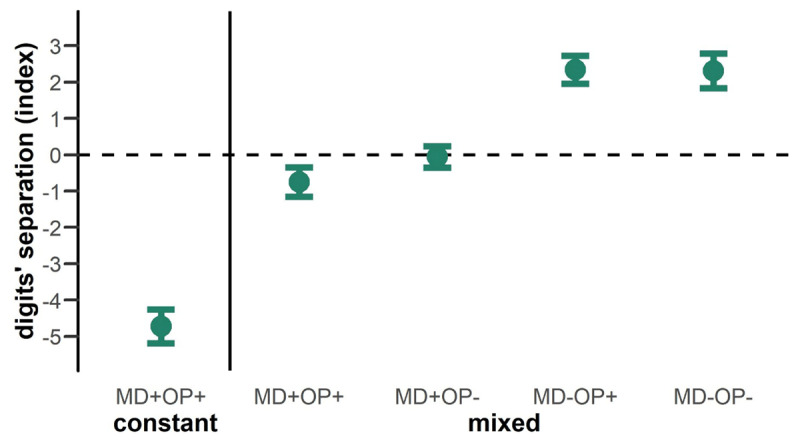
Mean values for digits‘ separation index. The values are plotted for the different configurations of feature repetitions with regard to the previous trial. Values for the constant and mixed part of the sequence are separated by the vertical line. Negative values indicate that participants positioned their digits in expectation of a repetition of MD that did occur, positive values also reflect this expectation, which, however was not met. Error bars display the 95% Cousineau-Morey within confidence interval.

*Mixing costs* appeared as expected. Comparing parts of the sequence where both object features were repeated (MD+ & OP+), the index value for digits’ separation was smaller during the constant blocks compared to the mixed block, *t*(30) = –10.12, *p* < 0.001, *d_z_* = –1.82 (Figure 2). In a situation where participants were sure to repeatedly grasp the same object, they were able to adjust their grasp so that it compensated for the MD experienced in the previous trial. As such, in digits’ separation a better performance was shown when object features were constant on a global level.

Within the mixed part of the sequence, participants also grasped the object in a way to compensate for the MD experienced in the previous trial, *F*(1,30) = 66.42, *p* < 0.001, *η*^2^*_p_* = 0.69, resulting in a mean index value below zero if the MD was repeated (*mean* = –0.41 ± 1.42), and in a mean index value above zero if the MD changed (*mean* = 2.33 ± 1.79). In contrast to MD, the OP alone had no influence on the index values in the mixed block, *F*(1,30) = 1.17, *p* = 0.288. However, we also found a significant interaction between MD and OP, *F*(1,30) = 11.13, *p* = 0.002, *η*^2^*_p_* = 0.27. The difference in index values between OP+ and OP- was more pronounced within trials with MD+ (*mean_diff_* = 0.70 ± 1.74) compared to MD- (*mean_diff_* = –0.03 ± 1.91), *t*(30) = 3.34, *p* = 0.002, *d_z_* = 0.60. In a situation with randomly mixed object features, the best performance was thus shown when both features were repeated.

### Object roll

**Figure 3:**
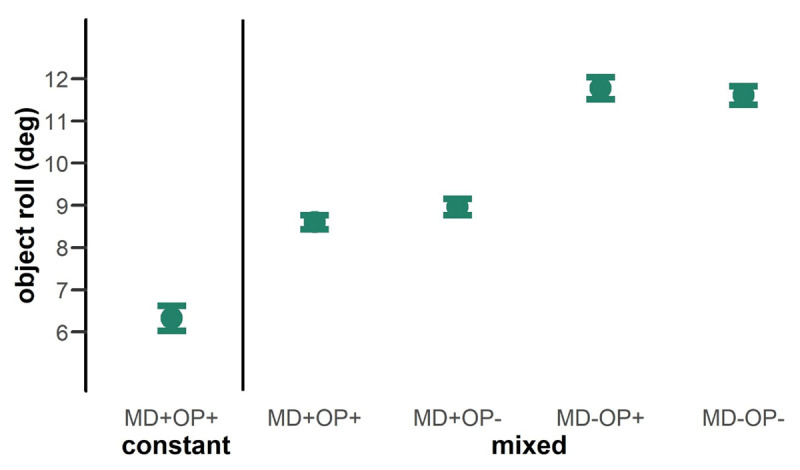
Mean values for object roll. The values are plotted for the different configurations of feature repetitions with regard to the previous trial. Values for the constant and mixed part of the sequence are separated by the vertical line. Values indicate the absolute maximal tilt angle of the object within the first 250 ms of object lift. Error bars display the 95% Cousineau-Morey within confidence interval.

Second, we investigated object roll as a measure of motor control processes. Since the positioning of the fingers strongly influences the extent of tilting of the object, mixing costs also showed up in the object roll, as expected. Comparing parts of the sequence where both object features were repeated (MD+ & OP+), the object rolled less during the constant blocks compared to the mixed block, *t*(31) = –10.65, *p* < 0.001, *d_z_* = –1.82 (Figure 3). When participants were sure to repeatedly grasp the same object, their compensatory grasp allowed them to perform a more stable lift so that the object rolled less in the first 250 ms. Thus, in object roll a better performance was also shown when object features were constant on a global level.

Within the mixed part of the sequence, the object rolled less in the beginning of the lift when MD was repeated, *F*(1,31) = 159.24, *p* < 0.001, *η*^2^*_p_* = 0.84, resulting in lower values for object roll (*mean* = 8.79 ± 3.39) compared to situation when MD changed (*mean* = 11.69 ± 3.48). The OP alone had no significant influence on object roll, *F*(1,31) = 2.97, *p* = 0.095, but we found a significant interaction between MD and OP, *F*(1,31) = 14.943, *p* < 0.001, *η*^2^*_p_* = 0.33. When MD was repeated, the object rolled less when also OP was repeated, compared to a change in OP (MD+OP+ < MD+OP-, *t* = –4.09, *p* < 0.001, *d* = 0.11). When MD changed there was no such difference (MD-OP+ = MD-OP-, *p* = 0.356). In a situation with randomly mixed object features, the best performance regarding the object roll was thus shown when both features were repeated.

### Correlation

**Figure 4:**
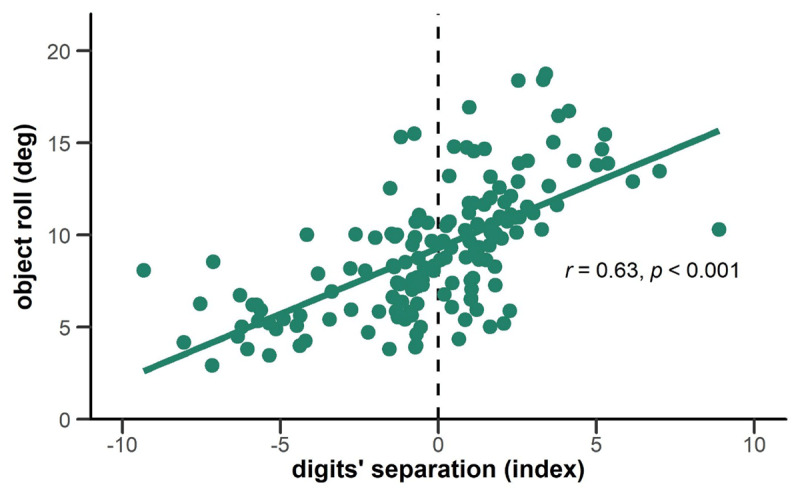
Correlation between digits’ separation and object roll. Values for digits’ separation are plotted on the x-axis, values for object roll on the y-axis. The two variables show a positive correlation.

Last, we analyzed the relationship between digits’ separation and object roll to test if more efficient grasping is indeed associated with less tilting of the object. We found a positive correlation between the two variables, *r* = 0.63, *p* < 0.001 (Figure 4). The object tilted less within the first 250 ms when the participants grasped the object more efficiently (reflected in negative values for digits’ separation).

## Discussion

In the present study, we aimed to investigate whether the principles of the BRAC framework, like binding and retrieval, also apply for more complex actions consisting of a sequence of discrete, goal-directed movements. Participants performed an unimanual object interaction task. They grasped an object and lifted it as straight as possible. In a sequential order, we varied two features of the object, its mass distribution and position. When both object features were constant on a global level, participants were able to adjust their grasp so that it compensated for the MD experienced in the previous trial, leading to a more stable lift and less object roll. Moreover, within a situation of randomly mixed object features, we found best task performance when both object features were completely repeated from one trial to the other. These results suggest that even in sequences of discrete movements features of that movement are stored in a common episode. Retrieval of this episode then influenced performance in subsequent actions. As these subsequent trial effects were present for digits’ separation and object roll they seem to influence both motor planning and motor control processes. To our best knowledge, this is the first time these basic principles of binding and retrieval, as described in the BRAC framework (Frings et al., 2020), were demonstrated in the context of more complex action sequences.

In general, grasping and lifting an object is a very familiar task we frequently perform every day. Therefore, it is not surprising that participants performed this task very well, especially in the constant blocks. When confronted with the same object configuration, they could effectively adjust their interaction with the object. This could explain their significantly better performance in the constant blocks compared to the mixed block in addition to short-term binding effects. In the mixed block, where the object configuration changed constantly, a stronger influence of the most recent experience can be assumed.

For both digits’ separation and object roll we found mixing costs comparable to those reported in previous studies using more artificial paradigms with simple motor tasks, such as single button presses (Los, 1999; Philipp et al., 2008; Rubin & Meiran, 2005; Steinhauser & Hübner, 2005). Participants were able to successfully adjust their lifting movements when they were sure that the object’s mass distribution or its position did not change from one trial to another. In comparison, the object tilted more when one of the object features could change in consecutive trials, although it did not. These results reflect the global expectation of consistency of the task and suggest that the object features and the action sequence were stored in a common episode. When the same object was presented in a consecutive trial, the action features were retrieved from memory, and thus significantly influenced the choice of action in the next trial. For the constant parts of the sequence, participants could be confident that object features would not change from one trial to another. In contrast, for the mixed part of the sequence, they always had to be prepared for the possibility that object features might change, which required additional processing to switch between the required actions which resulted in mixing costs. In contrast to typically considered temporal variables, such as reaction time, we were able to demonstrate mixing costs for kinematic variables, namely digits’ separation and object roll.

Retrieval of action features from memory has also been proposed in the context of grasp planning (Dixon et al., 2012; Rosenbaum et al., 2012). People grasp objects at a similar height as in the immediately preceding grasping movement, even when another grasp location would be more comfortable for the task at hand (Cohen & Rosenbaum, 2004). This grasp-height effect was explained by the re-use of the motor plan. A motor plan is generated in trial n which is then re-used in trial n+1 based on processes which retrieve the grasp location and not the grasp posture (Weigelt et al., 2007). Such retrieval of movement-relevant features is supposed to require the performance of the same movement under similar conditions within a short time frame. This account draws close parallels to principles of the BRAC framework with respect to feature binding and retrieval. To what extent both accounts relate to each other and could explain the various switch and repetition costs in movement planning and control remains a question for future research.

Besides these effects of global expectation, we found interaction effects of both object features for both kinematic variables within the mixed block of the experimental sequence, which are indicative of partial repetition costs. As shown in previous studies using button-response paradigms, participants usually perform better when the current features are completely repeated when confronted with a sequence of mixed stimulus-response combinations (Hommel, 1998, 2004). Repetition effects have also been shown in studies including reaching movement (Dixon et al., 2012; Randerath et al., 2015; Valyear et al., 2019). Here we were able to show a partial repetition effect for a sequence of discrete movements. In case of repetition of the mass distribution of the previous trial, partial repetition costs emerged if the object position was not repeated at the same time. Thus, in case of a repeated mass distribution, the object position appears to have been a crucial cue for retrieving the previous action from memory. In the case of non-repetition of the object position, there seemed to have been a conflict between the retrieved and the actually required action. Thus, especially in digits’ separation, there was no clear influence of the previous trial and participants tended to use a default grasp to lift the object. Even though within the mixed part of the sequence there was a general performance decrease when the mass distribution was not repeated. There was no significant advantage of a complete alternation of the two object features, as the BRAC framework would have suggested. Both digits’ separation and object roll were comparable irrespective of a change of the object position. There could be several reasons why this effect of a complete alternation did not appear in our results, such as the choice of kinematic variables, the complexity of the task, differences in the relevance of the object features, or even differences in the accessibility of object features throughout the trial.

Temporal parameters, such as reaction time, have been frequently examined in the context of the BRAC framework. These parameters are well suited for discrete actions, but less so for more complex action sequences, which involve a longer temporal structure than simple button-press responses. Thus, these temporal parameters might not be sensitive enough to capture binding processes within action sequences. Therefore, we chose to focus on kinematic aspects of motion, like the positioning of the fingers when grasping an object and the tilting of this object. These parameters are time-independent and at the same time capable to measure retrieval of previous actions. Previous studies investigating the influence of the general movement context and the feature configuration of the previous trial on movement kinematics have already demonstrated the potential of observing effects typically found in action control literature (Broda et al., 2020; Lukos et al., 2013; Voudouris et al., 2019). Additionally, it has been shown that an episode can persist for several seconds before losing their impact (Hommel & Colzato, 2004). Despite the fact that longer temporal distances between binding and retrieval of an episode tend to reduce their influence (Hommel & Frings, 2020), we observed binding effects in a grasping action that lasted about 5 seconds. Future research should examine how many motor features can be integrated into one single event file and whether and how this integration is influenced by spatial and/or temporal dependencies of the single action components (e.g., reach followed by grasp followed by lift).

The two object features, mass distribution and object position, did not seem to have the same relevance for the task performance. The object always had the same appearance (in terms of shape, color, texture, etc.), but could differ in its mass distribution and position. However, only one of these object features, object position, was visible at the start of each trial. The other feature, mass distribution, had to be experienced when grasping the object. Since the task was to lift the object as straight as possible and the mass distribution could vary at the same time, it seems reasonable that the mass distribution had a greater influence on task performance than the object position. This could explain why preceding actions were significantly influenced by the mass distribution and seemed to have been more decisive for the choice of action than the object position. In general, the position from which to grasp an object is not completely irrelevant. It needs to be considered when planning the grasping movement in order to be able to accurately reach the object. However, for the two performance parameters considered, digits’ separation and object roll, the mass distribution of the object still showed a stronger influence. It has been argued that task relevance is a crucial factor in binding features for action planning, and task-relevant features are more likely to become part of an episode (Memelink & Hommel, 2013; Mocke et al., 2020). More complex movements are particularly well suited to investigate features of varying relevance. Such actions offer the possibility to integrate several features in a natural way, not all of which have to be relevant for the task at hand. Future studies examining the BRAC framework could place a stronger focus on the varying relevance of features in order to map out a potential feature hierarchy to gain a more detailed picture of binding and retrieval processes.

Beyond differences in the relevance of the two object features, differences in their accessibility at the beginning of each trial could also explain the diverging effects. Usually, in paradigms investigating binding and retrieval, all features are perceivable throughout a trial. In everyday situations, however, we are also confronted with situations where object features are experienced during object interaction, as in our paradigm. Here, the object position was always visible, whereas the mass distribution could only be perceived by lifting the object. Following the assumptions of the BRAC framework, the retrieval of an episode should result in the retrieval of all object features bound within it and thus have an impact on behavior. Since participants always viewed the same object and performed the same task, we assume that both the object position and the mass distribution of the previous trial were retrieved from memory and consequently influenced their grasping and lifting behavior in the subsequent trial. Additionally, a temporal gradation of the retrieval process would also be conceivable. Retrieval of the object position was possible at the beginning of the trial, i.e. when this object feature was visible, whereas retrieval of the mass distribution could not occur before object lift. Therefore, our focus lay on measuring variables at a time when both features could be perceived and thus interact to influence the participants’ motor acts. Nevertheless, we cannot rule out whether the effects we found would change if the mass distribution had been visible from the beginning of the trial. Future studies could examine the role of temporally varying accessibility of features on binding and retrieval processes.

The task we used was considerably more complex and natural than previous tasks studying binding and retrieval processes in action control using single button press responses. It involved an object interaction embedded in an action sequence. However, the task did not involve a strong ecological relevance, as the object had to be lifted and placed back without a higher-level action goal. The present task allowed us to explore the usability of such a paradigm in the context of binding and retrieval and could stimulate further investigations using more complex action sequences. Future studies could create an even more natural context by assigning a higher-level goal to object interaction (e.g., drinking from a cup). For example, a (virtual) object might contain a liquid that should not be spilled during grasping and lifting. The effects found in this study might thus still be underestimated and become more pronounced with greater ecological relevance. Moreover, the BRAC framework should be tested for movements of higher complexity (i.e., more degrees of freedom) than the unimanual object interaction examined here.

## Conclusion

In summary, the results of the present study support the view that the general principles of action control postulated by the BRAC framework, such as binding and retrieval, appear to operate on a common, overarching basis. We showed that global consistency expectation and partial repetition costs affected how an object with varying mass distribution and position was grasped and lifted. These results suggest that even in more complex action sequences linked features are stored in a common episode and performance is influenced by retrieval of previous actions. Tasks such as these are thus capable of reflecting binding and retrieval and provide the opportunity of designing paradigms that are more natural and ecologically valid than previously used paradigms. Overall, this allows to compare and synthesize results on actions of varying complexity to provide a better understanding of action planning and control in everyday life.

## Data Accessibility Statement

The data collected for this work is publicly available at https://doi.org/10.17605/osf.io/ux5a7.
